# “Like filling a lottery ticket with quite high stakes”: a qualitative study exploring mothers’ needs and perceptions of state-provided financial support for a child with a long-term illness in Finland

**DOI:** 10.1186/s12889-020-10015-w

**Published:** 2021-01-25

**Authors:** Anna Paajanen, Kristi Sidney Annerstedt, Salla Atkins

**Affiliations:** 1 Department of Global Public Health, Karolinska Institutet, Solna, Sweden; 2 Global Health and Development, Faculty of Social Sciences, and New Social Research, Tampere University, Tampere, Finland

**Keywords:** Long-term illness, Disability, Child, Children, Parents, Finances, Financial struggle, Allowance, Support, Fatigue

## Abstract

**Background:**

A child’s long-term illness or disability is always a serious matter that impacts the whole family. Costs related to an illness can substantially affect a family’s financial situation. To date, there is little research on how parents experience available support for financial assistance. Surveys in Finland have found that families of children with long-term illnesses and disabilities could experience financial struggle and perceive the state provided financial support system as too complex. This article aimed to explore how caregivers of children with long-term illnesses perceived their financial situation, need for financial support and experienced its provision by the state in the Helsinki greater region.

**Methods:**

Convenience sampling was used. Participants were contacted through peer-support groups on Facebook. Eleven mothers of children with varying long-term illnesses and disabilities residing in the Helsinki greater region were interviewed using in-depth interviews. Recordings of the interviews were transcribed and analysed using framework analysis. An analytical framework was built to label the dataset, which was then charted. Lastly, themes were formed through descriptive analysis.

**Results:**

The main findings showed how the burden of caring for a child with a long-term illness or disability causes fatigue, which affects a family’s financial situation holistically. This affected both employment and financial management, but also receiving information about and applying for the state provided allowances. Mental resources were further depleted by seeking information and applying for allowances. This contributed to a vicious cycle between parental fatigue and financial struggle. Participants found the allocation of funds inequitable across the country. Finally, participants thought the allowance was insufficient in compensating for time spent caring for their child’s illness and did not consider their mental strain.

**Conclusions:**

Even in a welfare state such as Finland, caregivers of children with long-term illnesses are at risk of poverty and struggle with the organization of state provided financial support. Policies should be designed to ensure equity across the country and consider how the parental fatigue should be addressed. The study has implications for achieving sustainable development goals on wellbeing and reducing poverty.

**Supplementary Information:**

The online version contains supplementary material available at 10.1186/s12889-020-10015-w.

## Background

Globally, approximately 95 million children, 5,1% of all children, are estimated to be disabled [1]. The burden of long-term illness among children has grown proportionally the most in high-income countries [2]. Having a child with a disability^1^ or a long-term illness^2^ can bring financial difficulties to families. Although there is little research on how disability and poverty interact [6], those that do exist suggest that children’s disabilities are also linked to a risk of poverty [1]. Extra costs for caregivers relate to health care, therapies, mobility aids and disability equipment [6] but also transportation, clothing, laundry and special food [7]. In addition, often one of the parents, frequently the mother, reduces work time or takes sick leave [8]. Therefore, the longer a child’s illness lasts, the greater chances of parents’ income levels reducing [1, 8]. In order to deal with this income reduction, families might cut down the consumption of other goods and services [9], use more credit or receive help from relatives, friends or charities [10]. Should families struggle to make ends meet, the situation children can compound the effects of disability, through less emotional support [11], poorer health [12], undernourishment [13] or obesity [14] and poorer school performance [15].

In Finland between 15 and 30% of children are estimated to have a disability or a long-term illness [16]. The welfare state supports these families through a number of different benefits, including free healthcare for children under 16 years of age [17]. The Finnish social security institution Kela, together with the municipalities, offer social assistance through the two main social allowances, the disability and the caregiver allowance [18, 19]. A family can receive both these allowances. The grant is allocated according to the severity of the illness and need for care, regardless of a person’s financial situation [18]. For example 62% of those receiving the caregiver allowance work at least part-time, which is explained by the fact that in Finland all children are eligible for subsidised day care and free school [20].

To date, there has been little research in Finland on how families of children with disabilities or long-term illnesses fare financially, and deal with the financial support system. One survey, among parents (*N* = 189: 85% mothers) of children with diabetes, arthritis and asthma, found that a child’s long-term illness might cause financial struggle for families [21]. Another survey (*n* = 163: 91% mothers), pointed out that only about a third of children’s caregivers receiving the caregiver allowance felt that their family’s financial situation was good enough to cover all necessary costs and 9% reported not managing with their financial situation [20]. It has also been suggested that families do not know the municipal support system sufficiently and do not receive enough advice, resulting in problems in applying for and receiving the services [22]. There is, however, little understanding of the connections between the child’s illness, parental wellbeing, and financial situation.

It is important to ask whether families experience that the system is currently working well in ensuring financial security in case of a child’s illness, in order to ensure that the provided services are working as they should.

## Methods

### Aim

The aim of this study was to explore how caregivers of children with long-term illnesses perceived their financial situation, their need for financial support and how they experienced the provision of it by the state in the Helsinki greater region.

### Study design

This is a qualitative study using an interpretive approach [23], where participant’s experiences are central to the study. Qualitative methods were used in order to gain an in-depth understanding of parents’ experiences, and because the research team suspected another paper based survey would burden parents.

### Setting

The study took place in Helsinki greater region in Finland, which has approximately 25% [24] out of the country’s 5,518,000 habitants [25]. There are fourteen municipalities including the capital city Helsinki [24]. This means that the *caregiver allowance* as described in Table 1. is granted by many separate municipalities in this region [19]. The *disability allowance* is national [18] and everyone goes through the same application process.

**Table 1:** Disability and Caregiver Allowance

	DISABILITY ALLOWANCE	CAREGIVER ALLOWANCE
**Granted by**	Kela Finnish social security agency, national scheme [18]	Municipality [19]
**Who can receive**	When a child’s illness or rehabilitation needs last at least 6 months [18]	When a person needs caregiving at home due to a long lasting illness and a close relative or other is willing to give this care [19]
**Compensation level**	Basic support 92,13 €/month Raised support 215 €/month Highest support 416,91 €/month [18]	The monetary amount received differs within municipalities, the minimum amount nationally in 2019 is 399,91 € per month [26]. The sums change yearly and e.g. in 2012 the maximum amount varied between 728 and 1704 € [27]
**Recipients in Finland**	34,900 children received this allowance in 2017 [18] out of 948,404 children (under 16 years old) [28] Therefore 3,7% of all Finnish children received this allowance 40% of these receive basic support, 55% raised support and 5% highest support [18]	6700 parents of under 18 year olds received this allowance in 2014 [20] In 2014 there were 1,075,492 under 18 year olds in Finland [28], translating into 0,6% of all children receiving this allowance 91% of the people receiving this are women [20]

Helsinki greater region has more immigrants and young people in comparison to the rest of the country [25]. Its residents’ are wealthier and healthier than the country’s average [29]. Distances to social and healthcare services are relatively small [29], meaning that the transportation costs were not expected to climb high. However, compared to the rest of the country, the capital region has higher living costs, including cost of accommodation [25], which was expected to place a financial burden on the study population.

### Study participants

The study participants were adult caregivers of children (under 18 years of age) with long term illnesses living within the Helsinki greater region. The United Nations’ Convention on the Rights of the Child’s definition of a child as people under the age of 18 was used [30]. Convenience sampling [31] was used through eight peer-support groups on Facebook, intended for families of children with long-term illnesses. Convenience sampling was chosen as it was considered most feasible with a hard to reach group, who might experience considerable barriers to participating in interviews. The groups had between 816 and 4423 members at the time of the request. Fourteen mothers responded to the request for interview and 11 of them signed the informed consent form. Three people were not included as they lived outside the Helsinki greater region. Snowballing was attempted but participants explained that their peers were too exhausted to participate. Although there was no intent to only sample mothers, no fathers responded to the interview request. Participants were on average 38 years old (range 34–44 years). Most families had two children, with three having three children and one having one child. Two families had two children with long-term illness, others had one. Three of the respondents were single mothers, who were either students, unemployed or working part time. Two of respondents with partners were either stay at home mothers or on maternity leave. Four partnered respondents worked part time, and only two worked full time. Their children’s illnesses varied from neuro-psychiatric conditions such as autism and dysphasia to cancer and heart defects. Most children had multiple diagnoses.

### Data collection

Qualitative in-depth interviews [32] were used to collect data, which enable discovering each respondent’s individual experience and the meaning they ascribe to it, without imposing researchers’ views onto them. Interviews were conducted on separate days, during a four-week period in February and March 2019. All interviews were conducted and recorded in Finnish by AP, a researcher with a Nursing, Psychology and Global Health background. The interviewer had previously worked as a nurse with children who have long-term illnesses and disabilities and would often have lengthy discussions with their parents. These experiences resulted in comfort during interviews when discussing difficult and emotional matters.

Interviews were conducted at the participant’s homes or nearby libraries. The topic guide (See Appendix 1 in the supplementary file) was based on previous literature and expert consultation. It was piloted with a peer after which some repetitive questions were removed. The topic guide focused on perceptions of income and how the respondents felt their child’s illness affected their income, financial support from the state, application processes and how participants found information about them. A field diary was maintained after each interview to capture observations and reflections. The interviews lasted between an hour and an hour 45 min. The interviews were rich in content, as the participants seemed eager to tell their stories.

### Analysis

Framework analysis by Richie and Lewis [33] was used to analyse the data, which two of the team were familiar with (KSA and SA, both social scientists with global health interest and interest in social protection and health). The data analysis began during the interviewing process since interviews were transcribed simultaneously. Reoccurring concepts were noted during transcription and recorded in the field diary. The first two interviews were read by two independent researchers (AP; SA) and discussed. Codes were constructed based on the those interviews by AP and categorized to build an analytical framework [33] (see appendix 2 in the supplementary file for the theme index). The field diary was also consulted while developing the analytical framework. It was discussed among the team, revised and then applied to the entire dataset (i.e. labelled) [33]. The labelled data were then charted into a matrix [33] in Excel. While charting, the quotes were shortened with an effort to keep the participants’ own words.

During the descriptive analysis, data was re-read multiple times [33] both within columns and across participants. This allowed the range of perceptions and experiences of the participants to be detected and classified into themes and subthemes., which were translated into English. Theme development was discussed in detail with the multidisciplinary research team, and were revised following discussions. For example, some were merged to avoid repetition of the same content across themes. Discussions continued until consensus was reached. Finally, some central quotes were translated into English for reporting of the results.

## Results

### Overview of the participants’ financial situation

Most participants perceived that they managed well with their financial situation. However, a few lived solely on government support as they were a student or unemployed. Some reported struggling with everyday expenses due to low salary and compensating for it through using credit or foregoing essential items. These women described a constant fear of unexpected expenses. The costs associated with the child’s illness were identified as being related mostly to not being able to work, travel expenses to outpatient clinic appointments and to buying mobility aids and disability equipment. To compensate for the extra expenses, many participants received both the caregiver and disability allowance (as described in Table 1) from the government. Some participants only received the disability allowance.

We present the rest of the findings below (see Table 2 below for summary of themes).

**Table 2:** Themes and Subthemes

THEMES	SUBTHEMES
1. Caring for their child caused mental and physical exhaustion, which impacted the family’s financial situation negatively	1.1. The fatigue reduced a parent’s capacity to make money saving decisions
2. Working full-time and being a caregiver simultaneously was difficult, leading to a deterioration in a family’s financial situation	2.1. The fatigue made it more difficult to work or study 2.2. A parent’s time to work is limited due to their child’s care needs 2.3. Working life needs to be flexible for parents to be able to care for their child and work 2.4. Single parents experienced a larger financial burden when staying at home to care for their child
3. Participants found the application process for the state provided allowances as difficult and unjust	3.1. Information pertaining to allowances was not given through official channels and many parents did not have the resources to seek them out independently 3.2. The challenging application processes caused some parents to not apply for allowances even if in need 3.3. Parents felt that receiving allowances was unpredictable and lacked equity
4. While the social security support provided by the state was appreciated, the monetary amount was not enough for everyone	4.1. Finland’s social security system was seen as a privilege 4.2. The monetary amount of the allowance was perceived as ridiculous and the insufficiency led to some needing support from external sources

#### Caring for their child caused mental and physical exhaustion, which impacted the family’s financial situation negatively

Fatigue was raised in most of the interviews in one way or another. Many participants experienced their child’s long-term illness as a strain, as they used mental and physical resources to care for their child. In many families, the problems were seen during the day, as children needed more attention, care and guidance with everyday activities compared to a healthy child. However, many also reported the problems continuing during the night, as an illness might need monitoring or managing around the clock.

The participants reported this as resulting in fatigue, which presented as decreased energy and mental resources. This, in turn, seemed to affect the families’ financial situation in many ways. Fatigue thus became an overarching theme, and some aspects of it will be discussed under themes two and three, when discussing working life and applying for the allowance. The following subtheme 1.1. touches on how fatigue can have an impact on needing more financial support.

##### The fatigue reduced a parent’s capacity to make money saving decisions

Many participants did not save money for the future. Some reported trying but savings being redirected quickly to everyday necessities. Some described making decisions that saved other resources, such as time, rather than money, because of the child’s illness taking up resources:

*“I don’t have time to save. I don’t have time to make those foods that would save from our grocery expenses. … The resources just absolutely like aren’t enough. We don’t have time do go stroll in some flea markets looking for clothes that cost a couple euros. No chance.”,* Part-time entrepreneur, single mother, 2 children

#### Working full-time and being a caregiver simultaneously was difficult, leading to a deterioration in a family’s financial situation

Participants faced difficulties in working full-time whilst being a caregiver. Whether parents felt that they could work at all depended on how challenging they perceived the child’s illness to be. Most participants felt full-time employment was not conceivable while being a caregiver and only two worked full time. Apart from fatigue, the reasons for not working full time came down to needing someone to care for their child full-time or having to arrange meetings at outpatient clinics or the school during working hours. A few reported that if both parents work, though children attend daycare or school, the only option is to place their child in an institution. One family had made that decision, explaining that though they received professional support from the government for caretaking, it was not enough to allow both parents to work given the child’s appointments and needs.

##### The fatigue made it more difficult to work or study

Fatigue caused by the child’s illness was seen to affect the financial situation, as the caretakers were unable to work full-time. The families planned so that the illness would not affect their mental well-being too heavily, including taking extended periods of time off work, working part-time or not working at all. In most situations one parent reduced their hours, but some had decided that both parents would work less.

However, many described work as providing a welcome distraction from the stress at home. It was viewed as a mechanism to generate some of the previously lost mental energy:

*“Work has been an asset. It has been a place where you can go to and forget for a little while everything else that like is happening in this everyday domestic life”,* Part-time employed, 2 children

Many had decided to work part-time because they saw work as a positive influence. However, many also thought that the fatigue caused by their home situation affected their performance. For example, participants reported exerting additional effort to have productive interactions with co-workers. Fatigue also stunted innovation and development of their business as described below.

“*It became clear very quickly that I cannot for example develop my own business. Because my resources aren’t enough, because … my time and my energy and my brain activity is spent on keeping my son’s functional ability going”,* Part-time entrepreneur, single mother, 2 children

##### A parent’s time to work is limited due to their child’s care needs

The mothers described the extraordinarily large amount of time needed to care for their child. Health examinations, outpatient clinic visits and meetings at school were deemed the most time consuming and the main reason for not working. Some had several appointments a week, while others managed with yearly visits for evaluations of the illness. However, most had varying needs for visits without a clear routine to build work life around.

*“Eino’s rehabilitation meetings take up a lot of time. … There are anyhow sometimes several in a week. Occupational therapy, speech therapy, sometimes school meetings. Now there’s been umm meetings with social workers. Because we’ve been trying to get services already through the social side. Due to asthma there are doctor’s visits … due to [bedwetting] we have been to nurse’s and psychiatrist visits, evaluation visits, halftime meetings, everything possible. … Sometimes there are these weeks that I have three meetings, sometimes weeks that I don’t have any meeting.”,* Student, single mother, 2 children

##### Working life needs to be flexible for parents to be able to care for their child and work

Working part-time was considered a good option, bringing flexibility to manage making the care-visits. The participants working part-time discussed that if they worked full-time, they would need to take unpaid time off and financially the result would be the same. However, many thought that working life was not flexible enough to support combining the child’s illness with it. Often the decisions behind who would stay-at-home or work part-time would come down to whose work would allow it. For example, one parent might need to travel for work or shift work might bring more flexibility. Notably, in all cases it was the mother who decided to stay at home and in most cases the mother who had reduced work time.

Some had also stayed at home when maternity leave brought some flexibility, either after giving birth to a child with a long-term illness or a younger sibling at a time when their child fell ill. As maternity leave in Finland is longer than paternity leave, it was seen as only natural for the mother to stay at home.

As families weighed the options regarding caring for their child, some had decided to go back to or keep studying because that allowed them to care for their child more flexibly. One participant described in detail how working life does not consider the different needs that a parent with a child who has a long-term illness might have. For two of the participants, who were both single mothers, the lack of flexibility in working resulted in unemployment being a more fitting option.

##### Single parents experience a larger financial burden when staying at home to care for their child

One of the single parents described experiencing a larger financial burden due to not being able to work full-time and not having a partner who brings financial security. The importance of having two parents in case their child’s illness requires one to stay at home was further highlighted by participants who had decided to stay home and had a working partner. They felt that their partner being able to work was what initially made it financially possible for them to stay at home and be a caregiver.

*“I feel like I’m some sort of a free loader on the side of my husband. He feels that I make all of this possible”,* Stay-at-home mother, 2 children

Many mentioned that it was their partner who fully provided for the family financially and that without their income things would be a lot worse. Some partners also had a significantly higher than median salary, which contributed to the mother’s decision to stay at home. Some discussed the worry of being financially dependent on the working parent and what would happen in case of an accident, divorce, or pension.

#### Participants saw the application processed for state provided allowances as difficult and unjust

The third theme concerns how some parents did not apply for allowances that they were entitled to since they felt fatigued and did not have the energy to look for information or apply for the allowances. Most also described the process of applying as a burden that took up mental resources that they did not have. Some stated that applications themselves should be seen as work due to how much time and effort completing them took. Others described losing sleep over the difficult application processes.

##### Official information about allowances was not received and finding out about them alone required resources that parents did not have

It seemed that finding information about what allowances exist and how to apply for them was difficult. The combination of fatigue with challenges in finding information resulted in no capacity to even start looking for information about financial support, even if it was needed.

Some had received information and advice about the allowances from health care personnel or social workers, but they felt that their child’s illness severity meant that information was given more readily to them than others, resulting in an unequal treatment. Most reported that information was not given by the granting institution. Some felt that the granting institution made matters more difficult in order to save money:

*“So then no one gives you advice. No one ever says, especially from Kela, that ‘hey do you know that you’re entitled to this allowance’. So if you don’t happen to have a nurse, a doctor, a social worker, an occupational therapist who know about things. Someone who goes like ‘hey are you aware that’.. Or then a flock of friends, who have varying levels of special needs children, or a Facebook group for special needs children’s parents. There information is being shared and advice is given,- and instructions. But the party who grants the allowance, it really won’t help. It makes matters more difficult”,* Full-time employed, 2 children

Most participants reported finding information about allowances through friends or social media peer-support groups on Facebook, which were seen to give information quickly and easily. However, some also mentioned that the information received might not be correct.

##### The challenges regarding application processes led to some parents not applying for allowances even if in need

Once parents had information about allowances, they still needed to apply for it. The application process itself was also seen as a challenge, often described as “fighting”. Most participants felt that it was a time consuming and problems needed to be described very carefully in order to receive the allowances.

*“That disability allowance show … It’s this about four pages of explanations about all that he needs help with more than the other child and what all we use money on and how much and, and well where he goes for care and how he’s being cared for and once you’ve answered all these questions you continue with attachments … It’s really an extremely exhausting operation … It’s just horribly heavy. You have to write everything very precisely and, and like it’s just really very laborious”,* Full-time employed, 2 children

Some discussed having to write down everything based on the worst days and trying to represent their child’s illness or behaviour resulting from the illness as negatively as possible. Social workers or nurses who helped them fill applications also instructed them to do so. Dwelling on the worst days and focusing only on their child’s negative aspects when writing applications was seen to further worsen mental resources.

Criteria for the caregiver allowance were also seen as challenging, as they seemed to be made for elderly people, not children. Difficulties in applying for the disability allowance in turn were related to having to apply annually. This felt odd or even illegal to the participants because they did not believe that their child was going to get better. Nevertheless, every year they applied for the same allowance that helped them give their child the best possible care.

*“Even if it were just once a year but then there’s everything else. If you have some evaluation period twice a year and everything else in between then it’s like - ‘well, here we go again’…. Or it would be nice to know what would happen if all those resources that are used in the family (laughs) for applying for financial support, if they were used for supporting the child.”,* Unemployed, single mother, 1 child

Unfortunately, as mental resources reduced and the application process felt too difficult, some decided not to apply for allowances that they might have been entitled to. Some discussed knowing about certain allowances as their peers had received them, but when trying to understand why they had not applied for the same allowances it became clear that the problem was not having enough mental resources. Since they were exhausted, the applications were not filled, even if the money might have been necessary.

##### Parents felt that receiving allowances was unpredictable and lacked equity

Many participants felt that there were inequities across the country in applying for allowances, as the caregiver allowance is granted by municipalities. They felt that it would be better if the caregiver allowance was granted nationally, instead of locally, which could bring children to a more equal position.

Moreover, the participants felt that the granting process of the allowances was unpredictable.

*“It is a little like.. this kind of like filling a lottery ticket with quite high stakes”,* Student, single mother, 2 children

Participants reported that receiving the allowances depended on the officer who processed the application. Some had their allowances lowered or taken away, but they had no idea why. There was always the option to complain about the decisions, though participants felt that the officer responsible affected whether complaints were successful.

Finally, the participants brought up that they felt like the applicant’s writing skills brought children in an unequal position. The ones who felt confident in academic writing explained that they had no difficulty with the applications but were concerned for others.

#### While social security provided by the state was appreciated, the monetary amount was not enough for everyone

The final theme describes how the participants expressed mixed feelings about the allowances they received. A few had only negative things to say, while a few mainly brought up positive aspects. Most reflected on both the pros and cons of the Finnish social security system with regards to their child’s illness. Many reflected on how Finland has a great system on a global scale but also brought up their worries and frustrations about the system.

##### Finland’s social security was seen as a privilege

Many participants considered the almost free health care system as a financial benefit. They thought that the health care system was excellent, and that the medications were cheap.

*“Anyhow, like I said in the beginning that things are really good in Finland when we discuss like how at least I experience this care for long-term illnesses. Seriously when you think that the three months’ worth of insulin per kind is four fifty. It’s quite a small sum.”,* Part-time employed, 3 children

Some reflected on how they enjoyed free travel cards, cheaper swimming pool prices and events. These would bring monetary relief as families could enjoy doing everyday activities with less of a financial burden. Most, but not everyone, could enjoy these benefits, depending on their child’s illness.

##### The monetary amount of the allowance was perceived as ridiculous and the insufficiency led to some needing support from external sources

Only some of the participants felt that the amount received from disability and caregiver allowance was enough to cover the costs caused by a child’s illness. Most participants felt that the grant amount was simply ridiculous, and many felt that it did not even cover the costs related to their child’s illness. Participants thought that they should receive compensation for the hours spent tutoring and caring for their child, especially if the alternative was for the child to be in an institution where care would cost a lot more. One mother, who was unable to work due to the characteristics of her child’s illness, had calculated that her hourly wage for being a caregiver was 42 cents an hour.

Parents also felt that the allowances should consider the mental strain and fatigue that comes along with a child’s illness.

*“The criteria for children has been made for, for like congenitally disabled children. … But then it does not for example take into account the psychological strain that comes along with an illness that possibly leads to death”,* Part-time entrepreneur, 3 children

While most of the participants said they managed to pay for their everyday expenses with their income and the allowances, some struggled to the point of needing more financial help. Many participants discussed borrowing money from family and how that could either feel natural or excruciating and difficult to accept. However, some discussed the importance of third-party societal support like receiving food bags, food vouchers or help paying the bills from the church. While one participant felt privileged to be receiving help from the church another one felt it had been difficult to accept the help at first, as her family was not religious.

## Discussion

This study aimed to give the caregivers of children with long-term illnesses a voice in explaining how they perceived their financial situation, need for financial support and how they experienced the financial support system. The study found how the burden of caring for a child with a long-term illness leads to extreme fatigue, which affects a family’s financial situation holistically. Fatigue and the burden of care were reported to affect employment and financial management. Furthermore, receiving information about and applying for the state provided allowances was perceived difficult and unjust, and the monetary compensation seemed unfairly small.

### Disability, fatigue and poverty – a vicious cycle

Disabilities have been shown as a risk for financial struggle [13], which in this study is described as the need for financial support. Although many direct and indirect costs relating to children’s illness were identified, the greatest cost was seen as not being able to work full-time. However, in Finland 62% of children’s caregivers receiving the caregiver allowance work either full-time, part-time or are self-employed [20]. This is understandable as children’s dependence on parents vary, and as the participants suggested, work could be an asset that helped them cope with the difficulties experienced regarding their child’s illness.

**Fig. 1:**
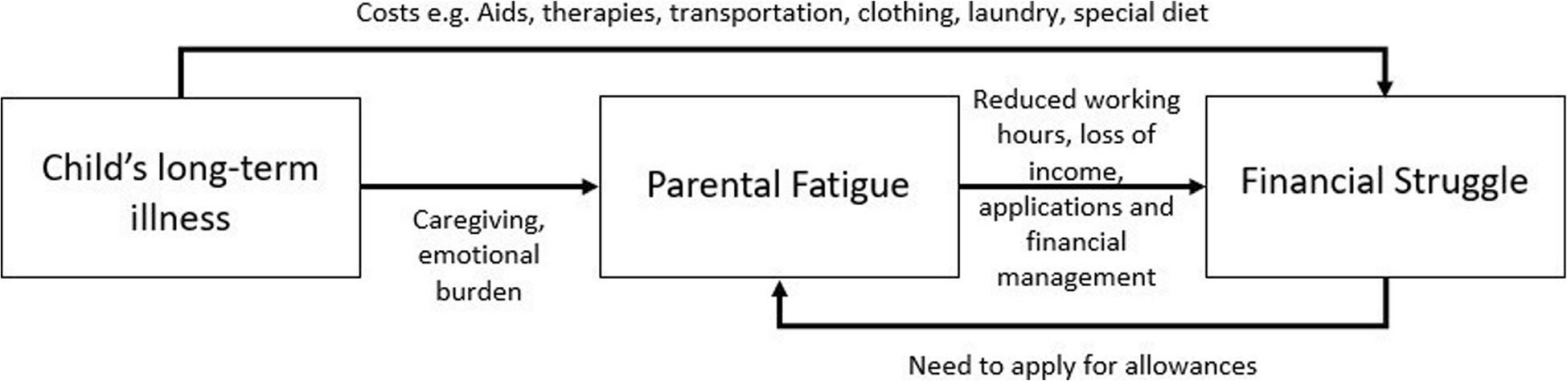
The interaction between a child’s long-term illness, parental fatigue and financial struggle

Although working was considered an asset, fatigue interfered with working full-time. The child’s illness can lead to a vicious cycle between parental fatigue and financial struggle illustrated pictorially in Fig. 1. As the child’s illness causes parental fatigue (i.e. stress and emotional pain), their mental and physical health deteriorates [34] leading to an inability to work full-time. Many caregivers do not know about existing services or how to apply for them [22] and looking for information on financial support might become too difficult especially given the above challenges. Although the Finnish law states that social service officials must give advice and counselling to people on the possible services and if needed, work together with the parties providing health counselling [35], the participants in our study reported not receiving official information about allowances.

Furthermore, once the information was found, studies suggest that the additional obstacle of applying for services could be too difficult to overcome considering the applicant’s current life situation [22]. This resonates with the findings of our study as mothers described how draining applying for the yearly disability allowance was. As the fatigue affected the participants’ lives holistically many hoped that the state provided allowances would take into consideration the mental strain caused by caring for an ill child. It might be justifiable to offer parents mental support for the fatigue without them needing a diagnosis or having to fight for mental health services. Availing mental health services for fatigued parents might improve their financial situation, if they then had more energy for work, financial management and application processes.

This study brings depth to understanding the interaction between disability and poverty, as it adds fatigue as a major contributor to financial struggles. Previous studies have looked at how a child’s long-term illness contributes to a risk of poverty [1, 8] and others have looked into how a child’s long-term illness or disability can lead to parent’s mental health deteriorating [34, 36]. This study found a connection, or rather a cycle, between parental fatigue and financial situation in a Nordic welfare state. Considering the definitions of disabilities and long-term illnesses it could be argued that fatigue is even a disability caused by a long-term illness. This would imply that a child’s long-term illness can also be disabling to the caregiver, which in turn is what affects a household’s finances holistically. This can result in a double risk of poverty, as not only does the child’s long-term illness have extra costs but the parent’s abilities to function in everyday life are further compromised.

### Mothers’ finances and their future

It is important to note that only mothers participated in the study. This is understandable, as in Finland 91% of children’s caregivers receiving the caregiver allowance are women [20]. The women in our study explained that their partner’s work either did not allow them to stay at home or that their partner earned more and staying at home would be a financial loss. This rings true when considering that men earn up to 16% more than women in Finland [37]. This raises questions about income inequality, and cultural and workplace norms about men staying at home to care for ill children. Some mothers mentioned that it felt natural to continue to stay home after their maternity leave ended. This is also in line with the Finnish cultural and statistical norm, as mothers are given more time off after childbirth than fathers [38]. The 4 month maternity leave [39] and the 6 month parental leave [40] is enjoyed in full by 90% of mothers [38]. However, the 2 month paternity leave [41] is not used by a third of fathers and only about one in ten use the parental leave [38].

Care burdens most often fall on mothers, affecting their personal finances [13], which is an important gender equality issue [42]. What is noteworthy is that a disabled child or a child with long term illness may get better or learn to manage their condition and move away from home (to their own home or to an institution) [20]. This was not discussed with the participants, nor did they bring it up when asked about the future. However, some participants expressed the worry of inadequate future pension and relying on their partners’ income. The risk for mothers who stay at home is that as they stay at home their knowledge regarding their field of work deteriorates and once their child moves away from home or gets better, getting back to work will be difficult [20]. As the risk of poverty can remain even after their child’s illness is gone or the parent is no longer a caregiver, it would be important to investigate whether the need for governmental support persists even after the caregiving duties are over.

### Implications of this study

This study contributes to the discussion about how long-term illnesses can cause financial hardship and how caregivers experience the Finnish social security system. As there is a social and health care reform ongoing in Finland, the families of children with long-term illnesses cannot be left behind. Participants were concerned that official information was not received about the allowances, applications processes were difficult and some needed additional support to meet everyday expenses. Furthermore, the emotional burden resulting from a child’s long-term illness should be considered when planning services.

Finally, based on the participants accounts it is suggested that the caregiver allowance should be made a national scheme in order to ensure equity for children around the country. The application forms should take into consideration the different nature of children’s and adults’ care needs. Moreover, the forms should be revisited to ensure that education levels don’t affect receiving grants. Furthermore, disability allowances should not need yearly applications, when it is known the situation will remain unchanged. Finally, both the caregiver allowance and the disability allowance need consider the mental strain caused by having an ill child and its implications on a family’s financial situation.

### Limitations and strengths

Time constraints and respondents’ fatigue made recruitment difficult, limiting our sample size. Originally purposive sampling was to be used, but the limited volunteers and time did not allow for this. Nevertheless, as at the eleventh interview data was considered saturated, this could be a minor limitation. Sampling participants through Facebook may have influenced their reports of where they found information the easiest. There may also be a gender imbalance on the peer support groups on Facebook, resulting in only women replying. However, it is very likely that only women answered as caregiver duties mostly fall on mothers.

Furthermore, it was communicated to the participants that the interviewer was studying Global Health and that her background was in nursing. This could have affected the way some participants reflected on matters and gave them preconceptions on what the interviewer knew.

The interviews had a sense of comfort as the participants could choose the place where the interview was held. Furthermore, all data were gathered by the same interviewer during a short period of time and the same person conducted the data analysis straight after the interviews. The interviewer also had a good understanding of the topic that was discussed and the culture in this region. During analysis, the original words of the participants were maintained for as long as possible, and the Finnish language was kept throughout data analysis. This ensured better quality of the analysis, in order not to lose meaning in translation. During the research process the interviewer discussed matters with the multidisciplinary team to increase confirmability and thick description to increase dependability of the study. Qualitative methods overall allowed us a deeper understanding of the issue at hand.

## Conclusions

This study brings depth to understanding the interaction between disability and poverty as it emphasises how a child’s long-term illness causes a vicious cycle between parental fatigue and financial struggle. Parents tended to have less energy and time to make money saving decisions or work full-time in order to receive an adequate income. Fatigue also affects finding information about and applying for state provided allowances. The application processes were seen as difficult, unpredictable and inequitable. Participants felt that the monetary amount received did not compensate for the time spent applying.

All these issues contribute to caregivers’, who are mainly women, wellbeing and risk of poverty. This issue requires further study to understand, for example through register-based studies on future outcomes for parents and the children. Policies should be designed to ensure equity, in Finland and elsewhere, and consider the different nature of children’s and adults’ care needs in application forms.

## Supplementary Information

**Additional file 1.**

## Data Availability

The datasets generated and analysed during the current study are not publicly available due to the sensitive nature of the data.

## Abbreviation

Kela: Kansaneläkelaitos. Finnish Social Insurance Institution of Finland

## Footnotes

1 United Nation’s convention for disabled people defines a person with a disability as those “who have long-term physical, mental, intellectual or sensory impairments which in interaction with various barriers may hinder their full and effective participation in society on an equal basis with others” ([3] , p. 14)

2 Long term illness is defined as a health condition that requires monitoring by a health professional and lasts at least 6 months [4]. Many long-term illnesses lead to disabilities [5], but it should be noted that many conditions fit under both definitions
