# Supplementary material for: “Like filling a lottery ticket with quite high stakes”: a qualitative study exploring mothers’ needs and perceptions of state-provided financial support for a child with a long-term illness in Finland
Source: BMC Public Health. 2021 Jan 25;21:208. doi: 10.1186/s12889-020-10015-w (PMC7830820; doi:10.1186/s12889-020-10015-w)
Supplement: Supplementary file 1 — Additional file 1. [file 12889_2020_10015_MOESM1_ESM.docx]

**Supplementary file: Appendices 1-3**

**Appendix 1. Topic Guide**

(Start by getting comfortable. Then once gotten comfortable, restate the purpose of the study, reaffirm confidentiality, ask for permission to record the interview and finally signature for informed consent form)

**Personal**

- Age
- How long have you lived in this area?
- What is your current daily activity? (work/studies/stay-at-home)
  - →*prompt*: How long have you worked/studied/stayed-at-home for?

**Family**

- Can you tell me about your family?
  - → *prompt*: Who is part of your family? Who do you live with?
  - → *prompt*: How old are your children/is your child?
- Can you tell me about any other relatives or close friends around?

**Child’s illness**

- Can you tell me about your child’s illness?
  - →*Prompt*: How and when did the illness start? Has the illness changed within the years?
  - → *prompt*: How does the illness show?
  - → *prompt*: What kind of an illness/diagnosis have they got?
- What care needs does the child have? (age-related and illness-related?)
- How is the child’s care organised?
- Can you describe your support system for caretaking?

→*Prompt*: For example, are there family members, friends, municipality employees that you can share the care with?

- How has the child’s illness affected your family?
  - Can you describe the challenges regarding your child’s illness?
  - →*Prompt:* How has it affected you emotionally and physically?

**Family finances and child’s illness’ impact on it**

- How is income derived in your family?
  - What thought does your level of your income bring up?
- Are there ways in which the child’s illness affects your family’s finances? How?
  - - → *prompt*: any income that would be generated in case of no illness?
    - Have the finances affected how your child’s care is formed? How?
    - For those who do not work, work part-time, have had time off before: how did the decision come about in the family for you specifically to stop working?
    - -> prompt: How does is affect your quality of life?

**Perceptions of financial situation**

- What costs are involved in caring for your child?
  - **→** *prompt:* how much do you feel this differs from bringing up a child without a chronic illness/disability
  - **→** *prompt:* Are there any direct costs such as medication, physical aids or indirect costs such as driving to a hospital/therapy, time usage?
- Does your financial situation limit you from purchasing certain things? Could you tell me about this?
  - **→** Does your financial situation affect the care your child can get? Can you explain?
  - → *prompt*: Does your financial situation affect the care the rest of the family can get?
- What are your possibilities of putting money aside for the future?
  - **→** How do you feel about the future and retirement?
- What kind of feelings does money bring up in your family?

**Financial support**

- Can you tell me what kind of government allowances are available to families like yours?
- Can you tell me about your situation regarding financial allowances?
- If not when have you ever considered applying, why/why not? Why did you not receive it?
- →*prompt:* Are there any other reasons that you can think of for why you might have/might not have received it?
- Where do you find out about governmental financial support?
  - Can you describe the application process?
    - If never applied: -> *prompt:* What has contributed to you never applying? What feelings does the application process bring about?
    - If applied: →*prompt*: What feelings does the application process bring about?
- Do you know if other families in your situations have received the financial allowance from the government?
  - →*prompt*: How do you feel about the fairness of the system?
- Is there anyone else you receive financial aid from apart from the government?
  - → *Prompt*: friend, family?
  - → *Prompt*: Can you describe your experiences/feelings about asking and/or receiving the aid from other than government?

**Finally, wrapping up**

- If you could change the governmental system regarding financial assistance, how would you change it?
- Is there any other forms of support you feel are essential?
- Is there anything else you would like to tell me?
- Would you like to tell me how you feel about this interview and the experience of being interviewed?

**Appendix 2. Index:**

Building life around the child’s illness

- - Fitting working life around the child’s illness
  - Fitting the housing situation and the illness together
  - Caring for the illness (e.g. outpatient clinic visits, time used for care)
  - Other decisions (regarding illness)
  - Difference compared to a healthy child

The available support

- - Experiences on the societal support (financial and non-financial)
  - Feelings about the societal support (financial and non-financial)
  - Ideas on how to improve the societal support
  - Getting information about the societal support
  - Applying for support
  - Support received from relatives and friends

A child’s illness causes fatigue

- - The burden/binding of a child’s illness
  - Fatigue affects working life
  - Other things related to fatigue
  - Aspects that increase mental resources

Experiences on the family’s financial situation

- - Expenses related to a child’s illness
  - Family’s income
  - Family’s expenses
  - Taking a loan
  - One’s properties cannot be turned into money/Things relating to one’s properties
  - Thoughts on financial situation
  - Financial managing within the family
  - Skimping

Other

- - Thoughts on future
  - Past experiences effect on thoughts

**Appendix 3. Informed consent form**

**Information for interviewees –** Experiences of parents with a child with a long term illness of financial situations

There has been global research about how families with children with long term illnesses have a greater risk of financial distress and that the illness can influence their financial situation in many ways. The matter has received fairly little attention in Finland and it would be important to hear from parents their thoughts and perspectives on how things are here.

The purpose of this study is to explore parents’ experiences of their financial situation when they have a child with a long term illness. The research will be conducted through individual interviews in a calm place chosen by the participant in February-March 2019. One can take part in the interviews if one is a parent to a child with a long term illness. The illness should have been present for at least six months and the child must be under 18 years old. The interviews are confidential.

This study is part of a Master’s thesis conducted at the Karolinska Institutet Global Health Master’s programme. The study has been designed by Anna Paajanen together with her supervisors (Salla Atkins and Kristi Sidney Annerstedt), and the study has no funding.

**The purpose of the data collection, its management and storage.**

The interviews are meant for Anna Paajanen’s Master’s thesis. The team also plans to publish an article based on the thesis in a suitable journal.

The interviews will be stored on a password protected computer in an access monitored room. The interviewer is the only person who accesses the recordings. She will transcribe the interviews into text, at which point the names of the interviewees and the names mentioned will be changed. As necessary, place or other names (e.g. workplaces) will be changed. The recordings and text files will be stored in a password protected file. Contact details will be kept separately from other information, also password protected. After the research the recordings and contact details will be destroyed.

**Benefits and harms of the research to the participants**

The interviewees will not receive financial benefits for taking part in the research. Going through experiences can be a positive experience, but may also cause anxiety. In these situations participants can contact the research team. The participants receive both the interviewer’s, and her supervisor’s contact details during the interview.

**The rights of the interviewees**

Participating in the research is voluntary. You may refuse to participate or withdraw from the study at any point. Your personal information will be maintained confidential. You may request information about the research at any point during the research.

**Contact details**

Anna Paajanen [anna.paajanen@stud.ki.se](mailto:anna.paajanen@stud.ki.se)

Salla Atkins [salla.atkins@tuni.fi](mailto:salla.atkins@tuni.fi)
